# Challenges and Achievements in Prevention and Treatment of Smallpox

**DOI:** 10.3390/vaccines6010008

**Published:** 2018-01-29

**Authors:** Sharon Melamed, Tomer Israely, Nir Paran

**Affiliations:** Department of Infectious Diseases, Israel Institute for Biological Research, P.O. Box 19, Ness-Ziona 74100, Israel; sharonm@iibr.gov.il (S.M.); tomeri@iibr.gov.il (T.I.)

**Keywords:** smallpox, vaccine, vaccinia, postexposure, MVA, LC16m8, Cidofovir, Tecovirimat, VIG, poly(I:C)

## Abstract

Declaration of smallpox eradication by the WHO in 1980 led to discontinuation of the worldwide vaccination campaign. The increasing percentage of unvaccinated individuals, the existence of its causative infectious agent variola virus (VARV), and the recent synthetic achievements increase the threat of intentional or accidental release and reemergence of smallpox. Control of smallpox would require an emergency vaccination campaign, as no other protective measure has been approved to achieve eradication and ensure worldwide protection. Experimental data in surrogate animal models support the assumption, based on anecdotal, uncontrolled historical data, that vaccination up to 4 days postexposure confers effective protection. The long incubation period, and the uncertainty of the exposure status in the surrounding population, call for the development and evaluation of safe and effective methods enabling extension of the therapeutic window, and to reduce the disease manifestations and vaccine adverse reactions. To achieve these goals, we need to evaluate the efficacy of novel and already licensed vaccines as a sole treatment, or in conjunction with immune modulators and antiviral drugs. In this review, we address the available data, recent achievements, and open questions.

## 1. Human Smallpox

Smallpox was a human pandemic disease caused by *variola virus* (VARV), a virus species within the genus *Orthopoxvirus* of the poxvirus family. Throughout the history, smallpox caused devastating pandemics that affected the world population. It is estimated that until the 18th century, around one person out of ten died of smallpox. Following a worldwide vaccination campaign launched by the World Health Organization (WHO), the naturally occurring smallpox has been eradicated [1,2]. In the following years, all known stocks of VARV were supposed to be destroyed or deposited in two WHO collaborating centers, in the United States and in Russia, that maintain and work with VARV in defined research projects under Biological Safety Level 4. The strict limitations in VARV research, and the fact that VARV infects and cause smallpox disease only in humans, limits our knowledge about the molecular mechanisms of smallpox pathogenesis, potentially complicating the development and approval of new effective antivirals and vaccines. Along with the successful eradication campaign, vaccination with vaccinia virus (VACV) against smallpox gradually discontinued. A consequence of this is that a growing part of the world’s population is not protected against smallpox and other orthopoxviruses. Recent assumptions that VARV or another pathogenic poxvirus, natural or synthetic, might be used as a bioweapon, or accidentally released from a laboratory [3,4,5,6,7], raise the awareness that human poxvirus infections might reemerge, resulting in an increased interest in the disease and its countermeasures.

Nowadays, after smallpox has been eradicated, and a large portion of the population is unvaccinated, several considerations should be taken to achieve effective management of an outbreak. (I) The exposure status—exposed individuals might be symptomatic or asymptomatic (incubating). Due to the extended incubation period, the ability to protect both exposed asymptomatic and unexposed individuals would require mass vaccination to ensure protective immunity. While vaccination is not useful for symptomatic individuals, other measures might be useful. (II) Efficacy of postexposure treatment—following infection, VARV evades the host immune system, contributing to disease severity, thus interfering with the induction of protective immunity by the given treatment (e.g., vaccine or immune stimulator). (III) Drug and vaccine availability—the development and approval of additional countermeasures, such as highly attenuated vaccines and antiviral drugs, and the ability to combine therapeutic modalities, opens new therapeutic avenues. Combining available data on the disease, countermeasures efficacies, challenge strains, animal models, correlates of immunity and experimental protection data, allows to recommend therapeutic approaches to control future outbreaks.

## 2. Human Infections by Orthopoxviruses

Viruses of the family *Poxviridae* contain large double-stranded DNA genomes of 130,000 to >300,000 nucleotides, and infect vertebrate (*Chordopoxvirinae*) and insect (*Entomopoxvirinae*) hosts. Humans can be infected with various poxviruses from the genera *Orthopoxvirus*, *Parapoxvirus*, *Yatapoxvirus*, and *Molluscipoxvirus*. VARV, the causative agent of smallpox, and molluscum contagiosum virus, are orthopoxviruses that exclusively infect humans. Similarly, ectromelia virus (ECTV) and camelpox virus (CMLV) also exhibit host restriction to mice and camelids, respectively. Other orthopoxviruses species, including VACV, cowpox virus (CPXV), and monkeypox virus (MPXV) exhibit broader host specificities, and occasional human infections results from zoonosis [8].

### 2.1. Variola Virus (VARV)

VARV is highly contagious and virulent to humans, and the estimated human lethal dose is 1–10 pfu. The obligatory infection of human hosts, together with the efficient protective immunity acquired by immunization, were the prerequisites for the successful eradication of VARV without the need to deal with natural reservoirs. Smallpox is a systemic febrile disease with typical rash, and a mortality rate of about 30% following natural infections. Both upper respiratory tract and skin/contact infections of humans with VARV are preceded by a rather long incubation of about 14 days (7–17 days). Following a sharp transient increase in body temperature and other symptoms, including backache, headache, vomiting, and prostration, centrifugal and synchronized rash develops throughout the skin, developing from macular to papular rash. The synchronized rash serves as a first diagnostic marker of smallpox and human monkeypox (MPX), distinguishing it from other human diseases that involve rash. In most cases, after several days, the rash heals, leaving notable pox signs on the skin [1,9]. In about 90% of the cases (ordinary smallpox as categorized by the WHO), the mortality positively correlates with the rash extent (10% to 80% fatality rates, mean 30%). Other less common forms of VARV infection are hemorrhagic smallpox, peaking in about one week (100% fatality rate), and flat smallpox, a more slowly developing disease course with high fatality rates (>90%). As smallpox has been eradicated almost 40 years ago, before sufficient data was collected, the underlying mechanisms of morbidity and mortality, as well as the determinants affecting the development of the various forms of the disease, remain elusive. However, collection of data, mainly from animal models of poxvirus infections, highlight the roles of virus-induced immune modulation [10,11,12,13] and immune pathogenesis (“cytokine storm”) in disease severity [14,15,16].

### 2.2. Monkeypox Virus (MPXV)

Although *Monkeypox virus* (MPXV) is less pathogenic and less contagious to humans than VARV, this natural pathogen of African rodents can cause severe human disease, with up to 10% mortality. Unlike VARV, MPXV exhibits a broad species specificity, and can cause fulminant disease in various animal species, including dormice, squirrels, prairie dogs and non-human primates [17]. Outbreaks of human MPX, believed to be through zoonosis, were reported in Africa (1970–1986, 1996–1997, 2017) and in the United States (2003). The disease manifestations in humans include maculopapular rash, and the fact that MPXV shares high similarity with VARV in structure, genome sequence, and antigenicity, raises the chance that MPXV will be misdiagnosed as human smallpox. Due to its similarity to VARV and its ability to efficiently infect humans and cause significant morbidity and mortality, MPXV is considered a potential agent of bioterrorism [18]. Since the 1970s, concomitantly with the discontinuation of the smallpox vaccination campaign, reported monkeypox cases in humans increased [19]. This elevation in reported infections reflects not only the increased awareness and reporting, but also other factors, including waning immunity to VARV and MPXV.

### 2.3. Cowpox Virus (CPXV)

*Cowpox virus* (CPXV), is an orthopoxvirus endemic to Eurasia that can infect and cause disease in a broad range of host species, including rodents, voles, domestic cats, horses, zoo animals (elephant, rhinoceros, okapi, cheetah), and human (zoonosis). In comparison to other orthopoxviruses, CPXV genomes encode the broadest set of viral genes, that unlike the host-restricted members (VARV, CMLV, ECTV), might enable CPXV to more efficiently evade the immune system and to more easily adapt to different species [20,21]. Yet, the contribution of each of those regulatory genes to virulence in the different hosts is not fully understood. Human CPXV infections through contact with diseased animals are mostly confined to local skin or eye infections (often spread by autoinoculation). Nevertheless, generalized infections with fatal outcome can occur in immunocompromised individuals. Similarly to human MPX, the increasing incidence of human cowpox (CPX) cases reflects, most probably, the decreasing smallpox immunity in the world population.

### 2.4. Vaccinia virus (VACV)

*Vaccinia virus* (VACV) is the prototype orthopoxvirus mostly known through its use as the smallpox vaccine. Various strains of VACV have been used to vaccinate against smallpox (see below); other mouse adapted strains, such as VACV Western Reserve (VACV-WR) and VACV IHD-J, are virulent to mice [22]. VACV infects a wide range of hosts, including rodents (mice and rabbits), large mammals like cows, non-human primates, and humans [22,23,24,25]. VACV inoculation of unimmunized or poorly immunized healthy individuals, through vaccination, results in a typical maculopapular lesion, clinically termed “take”. Other exposures, mainly laboratory accidents, usually result in local infections of the target tissue, such as the skin or the eye. However, VACV infection of at-risk individuals (e.g., people with immune deficiencies or allergies like atopic dermatitis), to whom vaccination is contraindicated, may lead to severe disseminated disease, which might be fatal. In recent years, in Brazil, there is an increasing incidence of natural exposures to VACV [25,26,27,28].

The increasing incidence of orthopoxvirus infections (MPXV, CPXV, VACV) worldwide, most probably reflecting the diminished coverage of worldwide smallpox immunity, highlight the risk that VARV release would lead to a major smallpox pandemic, necessitating efficient preparedness and efficient postexposure countermeasures.

## 3. Smallpox Vaccines

Throughout history, smallpox caused devastating epidemics, resulting in millions of mortalities worldwide. In the 10th century, reports from China describe the first attempts to control the disease by immunization, by applying a scab material from VARV infected patients to the dermis of naïve individuals, a process known as “variolation”. The success rate of those practices is uncertain, yet development of smallpox as a result of the inoculation was reported [1].

In 1796, Edward Jenner suggested a possible link between the presence of skin and mucosal lesions on cows, and on the hands of their caretakers, and the low percentage of smallpox among those caretakers. By skin exposure of a young child (James Phipps) to liquid recovered from those lesions, he eventually showed that the child was protected from a subsequent challenge with VARV. This finding of cross-protective immunization among orthopoxviruses led to the invention of the first vaccine—the smallpox vaccine [29,30]. Whether CPXV or VACV or other orthopoxvirus were used by Jenner is unknown. Recent work, analyzing the genomic content of an historical vaccine stock, produced by the Philadelphia company H.K. Mulford (Philadelphia, PA, USA), dating back most probably to 1902, revealed that this vaccine strain shares the highest degree of similarity with horsepox. This recent achievement supports the role of horsepox in the evolution of smallpox vaccine and as an ancestor of the VACV lineage [31,32]. During the eradication campaign, VACV became the vaccine strain used in the massive worldwide vaccination campaign, coordinated by the WHO, that successfully eradicated smallpox [1]. Since then, several vaccines were developed [33,34], and representative vaccines of the various generations are listed below.

### 3.1. First Generation Vaccines

During the 20th century, several VACV strains with variable biological properties were developed, and served as first generation vaccines for immunization against smallpox. These viruses were replication-competent, with variable reactogenicities to humans. Vaccines based on VACV strains Lister/Elstree, New York City Board of Health (NYCBH), EM-63, and Tian-Tan were predominantly used during the smallpox eradication campaign, because of their higher safety record compared to other vaccines, such as VACV Copenhagen or Bern [1]. These first generation vaccines were produced by several countries on various tissues: e.g., Lister-based vaccines were produced on chick chorioallantoic membranes, while NYCBH was propagated on calf or water buffalo skin. Vaccine formulations also varied from wet frozen vaccines to dried stocks (e.g., Dryvax) [1,35]. Vaccine production gradually discontinued as the disease was eradicated. The feasibility of smallpox reemergence by intentional or accidental release was the driving force behind renewal of vaccine stockpiling, vaccination of first responders and laboratory personnel, and evaluation of postexposure countermeasures.

### 3.2. Second Generation Vaccines

Adaptation of modern guidelines for the manufacturing of vaccines for human use led to the development of second generation vaccines. Those vaccines (e.g., Elstree-BN produced from the Lister/Elstree strain by Bavarian-Nordic (Kvistgaard, Denmark), Germany and ACAM2000^TM^-produced from the NYCBH by Acambis (Cambridge, MA, USA) [36], utilize the same historical vaccine strains e.g., Lister/Elstree or NYCBH with defined manufacturing processes, like the replacement of infected calf skin in the former Dryvax vaccine with Vero cells, the use of a cloned virus, and the production in compliance with guidelines for good manufacturing practices (GMP) to generate ACAM2000^TM^. The alterations in manufacturing processes and guidelines were intended to improve several parameters, including homogeneity, lot-to-lot consistency, and to minimize the theoretical risk of contaminations with adventitious agents. However, the major advantages of these second generation vaccines is the fact that they are based on the same (Lister) or a very similar (NYCBH vs. ACAM2000™) virus strain that was used during the eradication campaign, and therefore, they have a proven record for efficacy against human smallpox. This feature clearly contributed to the approval of the second generation vaccine ACAM2000™ as substitute for the expired historical vaccine Dryvax. However, as the second generation vaccines utilize the same vaccine strains as the first generation vaccines, they share the same virus-associated risks of developing vaccine adverse reactions (e.g., neurovirulence, eczema vaccinatum, progressive vaccinia, etc.) [37,38,39]. The risk of severe adverse events associated with both first and second generation vaccines, and the need to ensure safe vaccination even to individuals with contraindications to the current vaccines, is the driving force behind the process of developing and approval of third and fourth generation vaccines.

### 3.3. Third and Fourth Generation Vaccines

Unlike the first and the second generation vaccines that utilized vaccine strains of VACV with approved efficacy in smallpox eradication, an obstacle in the evaluation and licensing of new vaccines is that smallpox disease in humans no longer exists, and VARV infects and causes smallpox disease only in humans. Efficacy of these newly developed vaccines rely on animal models with related viruses, and collection of immune correlates arising from clinical testing in humans. Third generation vaccines are based on live, but highly attenuated VACV, with established safety and immunogenicity records (e.g., strains MVA, LC16m8, NYVAC, and dVV-L) and fourth generation vaccines are represented by non-infectious subunit vaccines (DNA, protein, recombinant viruses and replicons). Listed below are representative third generation vaccines. For a complete list, see [33].

#### 3.3.1. Modified Vaccinia Ankara (MVA)

Modified Vaccinia virus Ankara (MVA) is a highly attenuated strain of VACV that was originally developed from the ancient VACV-Ankara strain by >500 passages in chicken embryo fibroblasts for use as safer vaccine during the last decades of the smallpox eradication campaign. MVA was administered to about 120,000 individuals in Germany until 1980, without significant adverse events [40]. Since then, the vaccination dose was elevated by almost 2 orders of magnitude, yet vaccine safety profile has not been hampered. The attenuation process of MVA resulted in several large deletions in the terminal parts of its genome, and in many point mutations in comparison to conventional VACV strains, affecting genes involved in immunomodulation and host-range tropism. As a consequence, and unlike VACV, MVA exhibits replication deficiency in most human cells, while viral protein synthesis is unimpaired. Consequently, in vivo inoculation of MVA results in a strong stimulation of innate host responses, followed by efficient induction of adaptive immunity. Due to its replication deficiency, MVA exhibits a very high safety profile in cases to whom the conventional smallpox vaccines are contraindicated. These include immune compromised, allergic, and individuals with acute peri/myocarditis [41,42,43,44,45,46,47,48]. MVA as orthopox vaccine has been shown to induce solid protective immunity against lethal challenges with VACV, CPXV, or ECTV in mice, and against MPXV in cynomolgous macaques [47,49,50,51,52,53,54]. The inability of MVA to replicate in most mammalian cells restricts its ability to induce a durable immune response comparable to that of VACV, and the current vaccination protocols for MVA requires a two dose regimen with an almost 2 orders of magnitude higher vaccine dose than the conventional vaccines. The currently commercially approved MVA based vaccine, Immvanex^TM^, is produced by Bavarian-nordic, and approved as a third generation smallpox vaccine for adults in Europe and Canada [40].

#### 3.3.2. LC16m8

LC16m8 is a Japanese cell culture smallpox vaccine strain originating from the VACV strain Lister. The vaccine was developed in Japan in the 1970s, aiming to produce a safer smallpox vaccine than the conventional Lister/Dryvax strains. Attenuation was achieved by multiple passages of Lister in primary rabbit kidney cells at low temperature. LC16m8 can replicate efficiently only in primary rabbit kidney cells, but not in Vero cells. Furthermore, it is a temperature-sensitive strain. Truncation of the B5R open reading frame resulted in a truncated B8 antigen, and inefficient production of extracellular enveloped virions (EVs) most important for in vivo dissemination [55,56]. Indeed, concerns about the vaccine’s efficacy were raised [57], yet, the data indicates that antibody response generated following vaccination with LC16m8 is capable of neutralizing extracellular virions through antibodies against other EV epitopes, yet at a lower efficiency than antibodies generated following conventional smallpox vaccination [58,59]. The efficacy of LC16m8 has been addressed in various animal models, indicating that LC16m8 induces robust poxvirus protective immune response equivalent to the conventional vaccines [60,61,62,63]. Unlike MVA, LC16m8 can productively replicate in a broad range of host cells, and the genome of the virus does not contain other known major alterations in comparison to non-attenuated VACV strains. The replication capacity of the virus increases the production efficacy and the antigenic mass being produced in vivo and presented to the immune system, a major advantage of live vaccines. Yet, the same feature might increase the risk of adverse reactions during mass vaccinations, raising the question of whether LC16m8 is safe enough for at-risk individuals. In the late 70s the clinical testing of LC16m8 in more than 100,000 children in Japan indicated only few mild adverse reactions and the vaccine was licensed in Japan in 1975 but was not used during the eradication campaign since routine smallpox vaccination was halted in 1976. Since 2001, vaccine production started in Japan and safety and efficacy studies resumed. These studies confirmed the efficacy of LC16m8 in induction of major reaction (“clinical take”), and adaptive poxvirus immune response comparable to that of conventional vaccines [59,60,64,65]. Further preclinical studies using immune compromised animals further substantiated the efficacy [66] and safety profile of LC16m8 [67,68]. However, the replication capacity of LC16m8 and the risk of reversion to a full-length *B8* gene product, renders it potentially less attenuated than MVA and potentially less safe for vulnerable subjects. As the incidence of severe adverse reactions is extremely low, even large scale clinical trials of naive, healthy individuals, might not reach the vaccination coverage to uncover such cases. [59,66,69]. Excluding those contraindicated immune compromised subjects until further data is available, and coordinating data collection to cover the remaining gaps of safety and efficacy in at-risk populations, based on the efficacy and safety data collected so far, the overall preclinical and clinical data support further evaluation of LC16m8 as a promising third generation vaccine.

#### 3.3.3. NYVAC

NYVAC is an attenuated candidate vaccine virus originally derived from the Copenhagen strain of VACV. It was generated by deletion of 18 non-essential genes suspected to encode viral virulence factors, aiming to generate a highly attenuated vaccine platform. The virus poorly replicates in murine and human cells, but can efficiently grow in some mammalian and avian cell lines. In the past years, NYVAC vaccines have also been evaluated as third generation smallpox vaccine. Preclinical evaluation of NYVAC by vaccination of immune-suppressed macaques, followed by boost with a replication competent vaccine (Dryvax), demonstrated induction of immune responses and the ability to control the replication of Dryvax in immunocompromised individuals. However, such prime-boost regime did not confer protection from subsequent MPXV challenge [42,70], raising concerns about the efficacy of the strategy of prime-boost vaccination in immunocompromised humans. Recent evidence from immunizations in humans suggest that NYVAC induces significantly lower levels of humoral immunity than conventional Lister or Dryvax vaccines, shedding light on the contribution of vaccine-expressing immune modulating genes to vaccine efficacy [71,72]. Additional alterations were implemented in NYVAC, attempting to achieve better immunogenicity while maintaining a high safety profile [73,74].

#### 3.3.4. dVV-L

dVV-L is a replication defective third generation vaccine candidate that was generated by genetic modification of the VACV Lister strain through deletion of the *uracil-DNA-glycosylase* (*UDG*) gene—an essential component in poxvirus replication [75]. Productive growth of dVV-L relies on cell lines capable of complementing the UDG function, bearing the advantage of reducing the risk for adventitious agents by using defined and approved cell lines. dVV-L induces immune response and protective immunity comparable to MVA, and a good safety profile in immunocompromised animals. Moreover, solid protection of mice against lethal challenges with CPXV or ECTV could be demonstrated [50].

#### 3.3.5. Fourth Generation Vaccines

In addition to developing live attenuated VACV vaccines, efforts were made to develop novel orthopox-specific subunit vaccines. These fourth generation vaccines comprise of few viral antigens as proteins or genes expressed from DNA, or recombinant viruses or replicons. Of several antigens that were investigated, four, namely B5, Ll, A33, and A27, were mostly studied alone and in combination. Their combination was effective in several animal models, including MPXV infected non-human primates [76,77,78,79].

## 4. Vaccine Potency

A hallmark of poxvirus replication, in vivo, is the formation of dermal pock lesions. Whereas infection with highly pathogenic species like VARV, MPXV, CMPXV, and ECTV is associated with disseminated lesions in their natural hosts e.g., humans, camels, and mice respectively, dermal infection with other orthopoxviruses like VACV and CPXV results, in most cases, with a single lesion at the inoculation site. In continuation with Jenner’s achievement and based on the supremacy of the dermis in induction of immunity, vaccination with the historical strains of VACV that serve as smallpox vaccines are conducted by dermal exposure. Several methods have been developed throughout the eradication process, but the bifurcated needle was and is still considered a major achievement [80]. This specially designed needle was developed not only to hold the small amount (approximately 0.02 mL) of vaccine suspension, but also to administer it intradermally by scratching or multipuncturing, to concomitantly achieve dermal sensitization and vaccine administration [1,81]. This method of vaccination with the bifurcated needle simplifies the vaccination process, ensuring safe and efficient mass vaccination, a prerequisite when vaccination of a large population at a short time is needed.

## 5. Correlates of Immunity

Vaccination with VACV results in the appearance of “clinical take”—the typical pustule at the site of vaccination that is still considered the hallmark of vaccination efficacy. This measure of efficient vaccination is simple and applicable also in a population scale scenario. The efficient eradication of smallpox by vaccination enabled correlation of vaccination efficiency and the protection efficacy of the vaccine strains available at that time. In addition to vaccine “clinical take”, neutralizing antibody titer appears to correlate well with “clinical take”, better than other methods available at that time (like hemagglutination inhibition), and is still being considered a sensitive and reliable method for efficacy testing and a reference for evaluation of additional immune parameters (e.g., binding antibodies by ELISA) [82]. The ultimate VARV neutralization test, although limited to only two labs approved to hold and work with VARV, enables confirmation of the therapeutic potential of new products (e.g., neutralizing antibodies, vaccines, etc.) [83]. The broad repertoire of immune response parameters, that includes analysis of T cell response, cytokine profiling, and analysis of innate immune responses, were developed after smallpox eradication, and thus were not correlated with protection efficacy. The lack of a reliable VARV animal model further complicates this issue. Nevertheless, the availability and use of the historical vaccine strains that have proven efficacy and established measures of protection, can be used in conjunction with reliable animal models, to approve novel vaccines.

## 6. Animal Models

As smallpox has been eradicated, regulation and approval of vaccines and drugs that cannot be field tested relies on the FDA “Animal Rule” (21 CFR 601.90). This rule guides that approval of such products should rely on several parameters, including the use of appropriate animal studies in more than one well-characterized animal species. Listed below are representative animal models and the viruses used [33,84,85,86].

### 6.1. VACV, CPXV, and ECTV in Mice

Since VARV infection is restricted to humans, approval of new vaccines relies on established animal models that use closely related orthopoxviruses and various hosts [8]. Mouse models were developed with various VACV strains, both vaccine strains, and mouse-adapted, more virulent challenge strains (e.g., VACV-WR, VACV-IHD-J etc.). Mouse infections with recombinant VACVs contributed significantly to the understanding of the role of specific viral genes in disease progression and in eliciting immune response [22]. Mouse models were also developed using CPXV, yet a relatively high infectious dose is required to achieve lethal dose (1E5 pfu is LD_50_ in BALB/c mice independent of the infection route), unlike VARV in humans. Ectromelia virus (ECTV), the causative agent of mousepox, is a natural mouse pathogen [87,88,89]. Several models of mouse ECTV were developed during the years that show high degree of similarity between mousepox and smallpox at various parameters, such as very low lethal dose (1–10 pfu = 1 LD_50_), relatively long incubation period, and immune modulation strategy that is specific to the host. These, as well as other parameters, make ECTV infection of mice an excellent surrogate model for human smallpox [53,89,90].

### 6.2. VACV and Rabbitpox in Rabbits

Models were also developed in rabbits with VACV and rabbitpox virus (RPXV) [91,92,93]. While VACV infection induces local response, RPXV is highly pathogenic and contagious to laboratory rabbits (1–10 pfu = 1 LD_50_). RPXV is not a natural rabbit pathogen, but was rather isolated in an animal facility in Rockefeller Institute in 1932 [94,95]. As this virus is not a natural pathogen, the mechanism of its virulence is unclear, due to the limited availability of immune reagents, and the complexity to conduct large scale experiments with rabbits in BSL-3 facility (to protect the animal facilities) experiments with RPXV are relatively limited. Furthermore, a very short incubation period (about 2–3 days following respiratory infection) and the lack of dermal lesions, hampers the use of this model for the efficacy evaluation of postexposure vaccination.

### 6.3. MPXV in Rodents and Non-Human Primates (NHPs)

Monkeypox virus (MPXV), is a select agent associated with occasional human infections, with as high as about 10% incidence of mortality and disseminated lesions. Various models have been established throughout the years in various hosts. Non-human primates develop severe disease with disseminated lesions and death, following respiratory infections, yet a rather high dose of MPXV (doses of >10^6^ pfu) is usually needed to produce acute severe systemic disease [96,97,98]. This potential disadvantage of the non-human primate model and the inherent complexities of working with NHPs led to the development of MPXV models in small animal species, including prairie dogs, squirrels, African dormice, Gambian rats, and selected mouse strains with attenuated immune response [17,99,100,101,102,103,104]. Beside the major importance of MPXV as a human pathogen that can be used to model the disease in NHPs, it has several limitations: MPXV has broader host range than VARV, and is much less virulent to the hosts than VARV or ECTV, requiring a relatively high inoculation dose to cause disease. Furthermore, the length of the incubation period restricts the effective therapeutic window, and the fact that MPXV is a select agent that requires a BSL-3 lab to work with, somewhat complicates its use as a surrogate model for smallpox.

### 6.4. VARV, CPXV, and Calpox in NHPs

Modeling smallpox in rodents by infection with VARV is limited by the exclusive human tropism of VARV, and even SCID mice do not develop disease following high dose of infection [105]. Non-human primate (NHP) infection with VARV requires an extremely high viral dose (about 1E9 pfu) administered intravenously and intratracheally to induce disease [14]. Thus, and as VARV is not accessible to most researchers, this model has not been used extensively. CPXV has also been evaluated in infections of NHPs, resulting in variable disease outcomes depending on the route of infection. While intrabronchial infection resulted in infection of the respiratory tract, as well as lymphoid and internal organs, aerosol exposure resulted in respiratory tract pathology and intravenous CPXV infection resulted in severe hemorrhagic-like disease [15,106,107,108]. So far the NHP-CPXV model has not been used for vaccine efficacy studies. A model of NHP (common marmoset) infection with calpox has also been developed [109,110,111], yet immunological analyses in this model awaits further development, and vaccine efficacy has not been documented yet.

Overall, no single model can address the various aspects of disease progression and vaccine efficacy in postexposure scenarios, and the delicate use of hosts and viruses is needed to overcome the limitations embedded in each of these models.

## 7. Antiviral Therapy

During the eradication campaign and more intensively since then, several antiviral drugs have been evaluated, for treatment of both systemic disease and vaccine adverse reactions. These included therapeutic antibodies and antiviral drugs [37,112,113,114,115,116,117,118]. Listed below are few clinically tested representative drugs.

### 7.1. Vaccinia Immune Globulin (VIG)

VIG, is an IgG preparation from human plasma of smallpox vaccine recipients, indicated for the treatment of smallpox vaccine severe adverse reactions, such as progressive vaccinia, eczema vaccinatum, severe generalized vaccinia, and severe inadvertent inoculation. Unlike old formulations (15% IgG) that were administered intramuscularly, current preparations (5% IgG) are administered intravenously (VIG-IV). VIG preparations rely on the presence of plasma from smallpox vaccine recipients, are highly expensive, and their availability is limited. The long and good clinical experience with VIG, and its FDA approval, makes this drug the first line in treatment of adverse reactions. Whereas the therapeutic value of VIG in animal models and in the clinic is solid [41,119,120,121], data of prophylactic VIG administration for the prevention of adverse reaction in at-risk vaccinees, for various reasons, is limited [122]. In the last years, several therapeutic antibodies were developed and evaluated in animal models as potential VIG replacements [80,123,124,125,126,127,128].

### 7.2. Antiviral Drugs

Since smallpox eradication, several antiviral drugs were developed and tested in the various surrogate models [117]. Based on their efficacy in controlling virus replication and spread in animal models, these drugs are given as a second line of treatment, in cases of vaccine adverse reactions (VIG is the approved first line of treatment). Since these drugs were never tested in treatment of smallpox, and due to their limited availability and high price, their value as countermeasures against smallpox for a large scale pandemic is questionable. Listed below are the two drugs that are at the most advanced stages of FDA approval for human use.

#### 7.2.1. Cidofovir

Cidofovir (CDV) (Vistide), produced by Gilead sciences (Foster City, CA, USA), a nucleoside analogue approved for the treatment of cytomegalovirus (CMV) retinitis in people with AIDS, efficiently blocks the viral DNA polymerase, prevents viral replication, and is efficacious in treatment of poxvirus infected animals [129,130]. Beside these advantages, major obstacles include the need for intravenous administration and the inherent renal toxicity of the drug. The development of the orally available derivative Brincidofovir (CMX001) by Chimerix (Durham, NC, USA), overcame the obstacles of renal toxicity and drug administration [93,131,132]. Brincidofovir is not yet approved by the FDA.

#### 7.2.2. Tecovirimat (ST-246)

Tecovirimat (ST-246) (SIGA) (Corvallis, OR, USA), is a small molecule inhibitor that targets the viral envelop protein F13, and prevents enveloped virus egress. This drug is efficacious in various animal models [116,121,133,134,135,136,137], and unaccepted toxicity was not reported. Yet, the risk of drug resistance following extended use exists. The drug is currently evaluated by clinical trials as part of the FDA approval process, and is stockpiled at the USA for emergency use.

The above drugs were used in the last years for the treatment of patients with severe adverse reactions of the vaccines. Yet, the concomitant use of these drugs, during the course of treatment, does not allow for the determination of their sole efficacy in those cases. Although the above data strongly support the use of these drugs for treatment of smallpox [138], it is important to clarify that their effectiveness in the treatment of smallpox has not been determined. Furthermore, in a case of smallpox, due to the long incubation period, treatment of only symptomatic subjects maintain the asymptomatic carriers as a reservoir for continuing the infection. On the other hand, treatment of unexposed is not only useless, it will not protect them from future exposure from the circulating virus. Thus, despite the significant value of the antivirals, efficient containment of smallpox would not be feasible without effective treatment of symptomatic subjects and extensive vaccination, regardless of the exposure history.

## 8. Attempts to Extend the Efficacy of Postexposure Vaccination

The feasibility of postexposure vaccination against smallpox is based on historical anecdotal data, and on recent publications using surrogate animal models [33,85,86,139,140]. The cumulative data, considering gaps arising from limited epidemiological data and the ability to correlate data from surrogate animal models, suggest that active vaccination up to 4 days postexposure is protective [53,85,141,142,143]. However, as already discussed, in a population scale, the unknown exposure rate and the limited supply, high price, and unproven efficacy, do not allow reliance on antivirals only, but to vaccinate the population and to consider the combination of antivirals in conjunction with the vaccine. This understanding led to evaluation of the effects of co-administration of vaccine and antivirals, aiming to further extend the therapeutic window of smallpox vaccine and to concomitantly reduce vaccine shedding from the vaccination site, and the rate and severity of the vaccine-adverse reactions. Addressing the issue of extending the therapeutic window, however, is hampered by inherent limitations of the available animal models, like MPXV in NHPs, RPXV in rabbits, and VACV-WR in mice—mainly the length of the incubation period and the virulence of the viruses to the tested animals, necessitating high infection dose to induce severe lethal disease. For example, in NHPs, a dose of 1 × 10^7^ pfu of MPXV is about 6–7 orders of magnitude higher than VARV to human, and the extremely short incubation period of about 2–3 days is much shorter than in the human disease (7–14 days). Using such rapid and severe models restrict the ability of the vaccine to induce sufficient immune response, required for postexposure protection [53,141].

### 8.1. Combining Vaccine and Antivirals

One attempt to improve and/or extend the efficacy of postexposure vaccination was to co-administer the vaccine with an antiviral drug, like VIG, CDV, or ST-246 (summarized in Table 1) [53,85,115,120,134,139,140,144,145]. Indeed, the intended use of these drugs is either treatment of vaccine-adverse reactions once they occur, or given concomitantly to prevent the adverse reaction in at-risk populations [122,146]. Additional studies demonstrated the advantage of co-administration of antiviral with the vaccine to reduce the vaccine reactogenicity and virus shedding, while maintaining vaccine efficacy. As for their use in containment of smallpox reemergence, the therapeutic potential of these antivirals in treating poxvirus infected animals is well established, and efficacy has been demonstrated by either single or repeated administration. Yet, as discussed above, they cannot replace the essential requirement of population scale vaccination to achieve effective treatment, containment, and finally, re-eradication of smallpox. Since the available antivirals cannot discriminate between the vaccine virus and the virulent virus, upon co-administration of a drug and the vaccine, the drug might also block the vaccine replication, and would potentially reduce its protective value. Current data indicate that if reasonable doses of antiviral therapy are given, they will not hamper the vaccines efficacy [115,120], but can reduce vaccine shedding and the risk of developing vaccine-adverse reactions.

### 8.2. Combining Vaccine with Immune Modifiers

An alternative approach to extend the therapeutic window of postexposure protection is based on the use of immune modifiers, and more specifically, adjuvants, to improve the immune response to the vaccine (Table 1). Unlike protein based or inactivated vaccines that rely on adjuvants and repeated dosing to drive sufficient immune response, live vaccines, and specifically VACV, the “gold standard” vaccine, replicate in the host cells and induce effective immunity, even after a single vaccination without adjuvants. In rapid pre-exposure vaccination, somewhat lower vaccine doses were sufficient to prime the immune response and to confer protection to the subsequent challenge, rationalizing protocols of dose-sparing regimens, especially for MVA [147]. Yet, when postexposure immunization is requested, optimal antigen presentation is crucial. Previous animal studies demonstrated that, in a postexposure scenario, higher vaccine dose conferred better protection [53]. Elevation of the vaccine dose might not be applicable with the currently available and approved vaccines, and attempts to increase the vaccine dose might not be easy to approve, and would humper the feasibility of vaccine stockpiling for a large population scale.

We have recently demonstrated in a mouse model, that co-administration of VACV and the TLR3 agonist poly(I:C) conferred protection from an otherwise lethal ECTV exposure [13]. Interestingly, postexposure poly(I:C) administration protected mice, even in the absence of vaccine, indicating that the combined action of poly(I:C) with the viral antigens of the virulent virus (e.g., ECTV) efficiently induced robust and protective immune response. Thus, poly(I:C) induced efficient immune response that controlled the infection with ECTV. While postexposure poly(I:C) was protective, pre-exposure poly(I:C) administration was useless. Concomitant administration of poly(I:C) with the vaccine not only maintained vaccination efficacy, but also extended the therapeutic window of postexposure vaccination [13]. Whether the combination of poly(I:C) with the vaccine will also reduce vaccine shedding and reduce the risk of adverse reaction can only be deduced, but awaits experimental support.

Other attempts to improve the efficacy of the vaccine are the introduction of immune stimulatory genes into the viral (vaccine) genome, such as the introduction of IL-15 or gamma interferon into the genome of vaccinia virus [148,149]. Despite regulatory issues that may arise prior to their clinical evaluation, no data is available as to their therapeutic potential in a postexposure scenario.

Thus, based on the available preclinical data, the ability to administer an antiviral drug or an immune modifier like poly(I:C) concomitantly with the vaccine, enables the improvement of the therapeutic effect of the vaccine, to extend the therapeutic window of the vaccine, and to maintain the vaccine’s ability to ensure protective immunity in the targeted population.

## 9. Conclusions

The challenge of postexposure vaccination against smallpox has been addressed by several labs, and at present, the data supports the feasibility of emergency/postexposure vaccination. The use of various surrogate models contributed to substantiating the anecdotal historical data, and served to evaluate the therapeutic potential of vaccines, antiviral drugs, and immune modifiers. The disease in these models simulate human smallpox in many aspects, but develop relatively faster than smallpox in humans. Thus, the effective therapeutic window in humans might be even longer. Having the historical protective vaccine strains, the successful vaccination method (skin scarification) and the correlates of protective immunity as gold standards with approved efficacy, suggests considering a combination of antivirals/immune modifiers and vaccine to ensure an effective and extended therapeutic window (Table 2). Nevertheless, the data collected so far (Table 1) demonstrates the feasibility of extending the therapeutic window of postexposure vaccination, and improving vaccine safety by co-administration of the vaccines with antivirals or immune modifiers. This would ensure effective treatment of exposed individuals while protecting the unexposed population from future exposures, allowing to control the infection.

## Figures and Tables

**Table 1:** Efficacy of postexposure combination therapy.

Treatment	Host (Pathogen)	Incubation Period	Therapeutic Value	Ref.
Vaccine	Human	7–14	Effective up to 4 days postexposure (anecdotal).	[85,139,140]
Vaccine (Lister, MVA)	Mouse (ECTV)	6	Effective up to 4 days postexposure (Lister 10^8^, MVA 10^8^).	[53]
Vaccine + VIG	Mouse (ECTV)	6	Maintains vaccine efficacy. Effective up to 3 days postexposure.	[120]
Vaccine (Lister, MVA) + CDV	Mouse (ECTV)	6	Maintains vaccine efficacy. Effective up to 3 days postexposure.	[115]
Vaccine + CMX001	Mouse (ECTV)	6	Effective 2 days postexposure (no data on delayed treatment). Maintains vaccine efficacy.	[145]
Vaccine + ST-246	Mouse (VACV-WR)	3	Maintains vaccine efficacy. Effective up to 3 days postexposure.	[134,144]
	NHP (MPXV)	2–3	Effective 3 days postexposure (no data on delayed treatment). Maintains vaccine efficacy.	[133]
Vaccine (Lister) + Poly(I:C)	Mouse (ECTV)	6	Effective up to 5 days postexposure.	[13]

**Table 2:** Approaches for prevention and treatment of smallpox.

Exposure Background	Appearance	Therapeutic Approach			
		Vaccine	VIG	Antiviral Drug	Immune Stimulator
Unexposed	Asymptomatic	+ ^1^			
Unknown	Asymptomatic (incubating)	+ ^2^			
		+ ^2^	+/− ^3^		
		+ ^2^		+	
		+ ^2^			+
Exposed	Symptomatic	− ^4^		+	
			+/− ^3^		

+ Effective/required; +/− limited; − not required/not useful.^1^—Prevention; ^2^—Vaccination required to ensure immunity. High dose recommended; ^3^—Limited supply, intravenous administration. Preferably for the prevention and treatment of vaccine adverse reactions. Improved efficacy by combination with antiviral drugs; ^4^—Vaccination not required for symptomatic people.
